# Predicting the conformations of peptides and proteins in early evolution. A review article submitted to Biology Direct

**DOI:** 10.1186/1745-6150-3-3

**Published:** 2008-01-28

**Authors:** E James Milner-White, Michael J Russell

**Affiliations:** 1 Institute of Biomedical and Life Sciences, University of Glasgow, G12 8QQ, UK; 2 Jet Propulsion Laboratory, California Institute of Technology, Pasadena, CA, 91125, USA

## Abstract

Considering that short, mainly heterochiral, polypeptides with a high glycine content are expected to have played a prominent role in evolution at the earliest stage of life before nucleic acids were available, we review recent knowledge about polypeptide three-dimensional structure to predict the types of conformations they would have adopted. The possible existence of such structures at this time leads to a consideration of their functional significance, and the consequences for the course of evolution.

This article was reviewed by Bill Martin, Eugene Koonin and Nick Grishin.

## Text

In the quest for the origins of life, bioscientists, retracing genetic steps, seek its source in an RNA world [1,2]. Considering the earliest, pre-RNA, period of evolution, earth scientists prospect for the free energies and the mineral catalysts that promoted reactions between oceanic dissolved CO_2 _and submarine alkaline hydrothermal effluents of H_2_, NH_3 _and CH_3_S^- ^issuing at ≤120°C into the inorganic pores of an ocean floor hydrothermal mound. It is suggested that a gradual organic takeover of inorganic, probably Fe > Ni sulfide, compartments at such a mound, was effected as carbon dioxide was reduced with hydrothermal hydrogen to simple organic molecules, including amino acids and short peptides [3-6]. That amino acids and peptides can be produced under the conditions expected at hydrothermal systems has been demonstrated experimentally [7,8]. As with other organic molecules, their concentrations are likely to have been concentrated by thermal diffusion with the inorganic pores occupying the mound [9]. Previously we pointed to the likely significance of nests (3–6 residue peptides whose mainchain NH groups bind anionic atoms or groups) at the earliest stage of evolution [10]. Here the entire range of possible polypeptide conformations are considered, focusing on those expected to have been common and to have encouraged such suboceanic hydrothermal systems to evolve.

**T**he multi-layer β-sheet of mature amyloid may have formed the material of the earliest organic membranes. There would have been a high proportion of enantiomeric polypeptides (where the main chain atoms of successive amino acids are enantiomers). Nests would have been prominent and used for binding phosphates, pyrophosphates and iron-sulphur centres such as the water soluble [Fe_4_S_4_(CH_3_S)_4_]^2- ^cluster assembled abiotically in aqueous media [11]. Their evolutionary relics are detectable in present day proteins [11-13]. Multi-layer α-sheet would have formed readily, possibly generating cation and proton ion channels at its margins. On the other hand, the proportion of α-helix and polyproline type II helix would have been lower than in present-day folded proteins. The different conformations are listed in Table 1.

**Table 1:** The major categories of protein conformations:

Conformation	Conf. type	Native folded	Native unfolded	Native unfolded and denatured.	Early peptides.
α-helix	Reg.rep.	+++			+
β-sheet (as in native proteins)	Reg.rep.	+++			++
PPII (polyproline type II)	Reg rep	++	+++	+	+
nests	Enant	++	++	+	+++
amyloid (multi-layer) β-sheet	Reg.rep.			+++	+++
(multi-layer)α-sheet	Enant.		+	++	+++
metal-peptide	Reg.rep.	+	+	+	++

Notes: The distributions of conformations in folded, unfolded and denatured native proteins are indicated and also their expected distributions in early peptides assuming they consist randomly of L and D amino acids and a high proportion of glycines. Enant. indicates enantiomeric peptides, where main chain atoms of successive amino acids are enantiomers. Reg. rep. indicates conformations where main chain atoms of successive amino acids are the same (the α-helix is right-handed in proteins; PPII is a left-handed helix in proteins; β-sheet has two forms with opposite handedness, one of which predominates in proteins, and, unlike the helices, intermediate or mixed forms are possible). In peptides with random proportions of L and D amino acids the two chiral forms are equally likely. Beta-sheet in native proteins has at most four layers of sheet and less than 300 amino acid residues, while amyloid and α-sheet have multiple layers and typically thousands of residues or more.

## Evidence for early peptide formation

The first sulfide to precipitate on the mixing of the HS^-^-bearing hydrothermal fluid and Fe^2+^-bearing ocean water upon the mound would be nanometric particles of the layered mineral mackinawite [Fe >> Ni > Co)S] organised as rather leaky membranes [4,14-17]. Glycines (NH_3_^+^CH_2_COO^-^) and other simple amino acids may have been condensed with pyrophosphate [3]. (Condensation with gaseous carbonyl sulphide has been demonstrated [18] although as COS is extremely unstable in alkaline solution [19] we do not consider it a viable mechanism). PPi, acting as the first condensing agent in place of ATP [20], could have been recharged variously by the natural protonmotive force operating across the inorganic membrane (pK_a_~9 at low water activity [3]), or through the reaction between acetyl phosphate and inorganic monophosphate in the mound [6,21].

The reductive amination of the appropriate α-keto acids, synthesised under simulated hydrothermal conditions, has been demonstrated [22]. Alanine, glutamate, phenylalanine and tyrosine yields of up to ~50% in the presence of freshly precipitated FeS at 50° and 75°C has been achieved [8]. It is notable that high amino acid yields required a pH of 9 ± 0.3, presumably because the uncharged form of ammonia acts as the nucleophile (pK_a _of ammonia = 9.25; [8]).

Peptides have been generated [7,23] by activating 500 mM of glycine, phenylalanine and tyrosine with CO at 1 bar with 1 mM of freshly precipitated (Ni, Fe)S, and methane thiol or H_2_S as catalyst at 100°C. Significant yields of glycylglycine (≤11%), phenylalanylphenylalanine (≤8%), and tyrosyltyrosine (≤3%) were produced in one day, also at a pH of ~9.1. A 6 ppm yield of tyrosine tripeptide was realized within 4 days. Finding that, as with their amino acid syntheses, alkaline conditions (pH range 8 to 9.5) were a prerequisite for high yields, solutions were buffered [23] with Mg(OH)_2_, a serendipitous choice since, as the mineral brucite (Mg(OH)_2_), it buffers off-ridge hydrothermal systems at high pH [16,24,25]. Although starting with enantiomerically pure (L) amino acids, both the phenylalanine and tyrosine dipeptides had epimerized to pure L, D, and mixed L and D dimers in a few days. Huber and Wächtershäuser [7] concluded that at the origin of life amino acids would be racemic.

Longer peptides have been produced abiotically, though so far in rather extreme conditions. For example, thermal cycling at pH 2.5 between 0 and 250°C oligomerizes glycine up to hexaglycine [26]. Also, oligomerization of glutamic acid has been effected using carbonyl di-imidazole as the condensing agent [27]. The synthesis of peptide polymers of up to 10-mer are possible in solution, but longer polymers of between 30 and 50-mer adsorb onto the required solid phase (non-catalytic) surface of montmorillonite, though this is a smectite clay mineral to be expected in alkaline hydrothermal submarine mounds [28]. Pressures up to 25 MPa (~250 atmospheres) are expected to encourage the rapid 2- to 10-mer polymerization of dry glycine at 150°C [29]. Ferris et al. [27] imagine mineral surfaces covered "by polymers of enormous length". Such peptides could contribute to the generation of protoenzymes as well as to membrane and cell wall structure of the earliest cells.

## Early polypeptide conformations

Here we consider the likely conformations of short polypeptides composed randomly of D and L amino acids and with high glycine content. To crystallographers, short polypeptides are notoriously structureless, in that they tend to flicker between conformations rather than staying fixed in one. It could be said they more often belong to the "natively unfolded" category [30] than native proteins do. However, many exhibit particular structures and this is what we shall consider. One categorization of relevance to early evolution is the distinction between regularly-repeating polypeptides and enantiomeric ones. This refers to the main chain atoms of successive amino acids, which have identical conformations in the regularly repeating category but are enantiomeric, meaning mirror images, in that category. Of course another category – neither of these – also occurs commonly but is not given a particular name.

### a. Regularly repeating peptides

**Figure 1:**
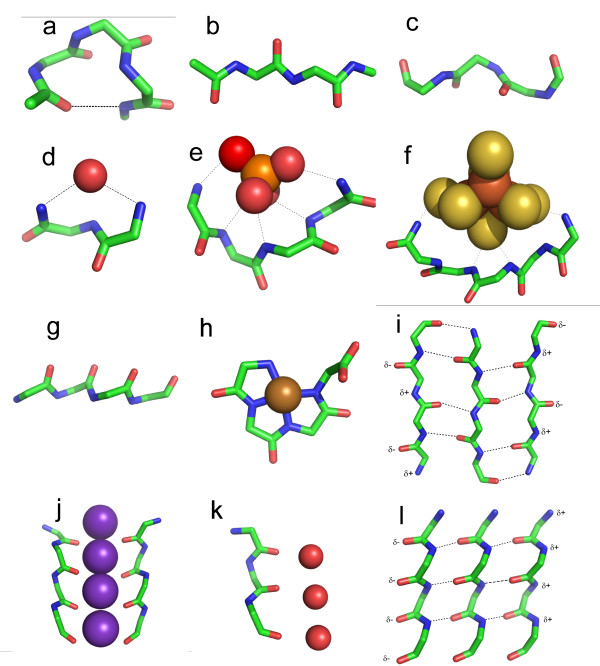
**Polypeptide conformations**. Stick pictures show main chain atoms of polypeptides; side chains are omitted; colors: carbon, green; oxygen, red; iron, rust; nitrogen blue; phosphorus, orange; sulphur, yellow; nickel, brown; potassium, purple. **a) **α-helix **b) **β-strand, as in **i c) **polyproline type II helix **d) **RL nest **e) **phosphate bound to LRLR nest **f) **Fe_3_S_4 _bound to RLRLR nest **g) **α-strand, as in **l h) **Nickel-tetraglycine [62] **i) **β-sheet **j) **A row of potassium ions bound at the selectivity filter of the potassium channel; only two of the four polypeptides are shown. **k) **A row of waters (hydrogens not shown) bound to part of the aquaporin channel **l) **α-sheet, made from strands as in **g**, a possible early membrane.

About two thirds of polypeptides in existing native folded proteins occur as α-helix, as in Fig 1a, or β-sheet, shown in Fig 1b. Native proteins also have a few percent of short pieces of polyproline type II helix (PPII; polypeptides with this conformation, in spite of the name, are not necessarily composed of prolines), as in Fig 1c. However, when it comes to unfolded native proteins, or to short polypeptides, evidence [31] suggests that PPII is the most common conformation. At first sight, it might seem likely that the earliest proteins would also tend to consist of PPII. However, the earliest peptides are expected to be made of mixtures of L and D amino acids [7]. In the same peptide, they would be incompatible with the chiral PPII helical structure. The same applies for chiral α-helix. This leads us to consider enantiomeric peptides, which tolerate, and can be favored by, such amino acid compositions.

### b. Enantiomeric peptides

In regularly-repeating polypeptides the main chain conformations of successive amino acids are the same, or at least roughly so. Polypeptides can also fold to form a different type of structure where the main chain conformations of successive amino acids are enantiomers (mirror images). The φ, ψ values of one amino acid (the φ, ψ torsion angles, measuring rotation about the N⋯Cα and Cα⋯C bonds, are major determinants of polypeptide conformation) are equal to the φ, ψ values of its neighbour multiplied by -1. Such polypeptides [32,33] are achiral and without pitch, forming rings or chains, but not helices, as in Figs 1d–g and 1j–l; they are expected to be favored by achiral or alternating racemic amino acids. This may be contrasted with the various chiral helices (strands of β-sheet are geometrically types of helix) observed in modern proteins with mainly chiral amino acids. Enantiomeric polypeptides occur as two kinds, nests and α-sheet, described below.

### c. Nests

Nests are motifs made from 3–6 amino acid residues where successive main chain NH groups bind anionic atoms or groups [34-39]. One with 3 residues is shown in Fig 1d. Currently, 5–8% of residues in proteins belong to nests [34,36]. Their residues have main chain φ, ψ conformations alternating between about -90°, 0° and +90°, 0°. In most nests an anionic atom or molecule is firmly hydrogen bonded to the NH groups of residues i and i+2 and less strongly or hardly at all to the i+1 NH group, which often points slightly away. In typical present day nests glycine is preferred at alternating residues resulting in a conformation where the anion is partially encircled. This is because glycines are achiral, and can adopt the φ = 90°, ψ = 0° conformation favoured by D-amino acids equally with the φ = -90°, ψ = 0° one favoured by L-amino acids, i.e., they can behave like the D enantiomer. As glycine was probably the main amino acid generated within the hydrothermal reactor [40], it is likely nests were more abundant in early peptides than in present day proteins. But equally the synthesis of alternating L-amino acid/glycine, D-amino acid/glycine or L-amino acid/D-amino acid peptides [41] in the mound would give rise to nest conformations with anion binding capacity.

The clue as to how inorganic phosphate was first ligated is given by the P-loop, the commonest and best known of the protein motifs that bind ATP/ADP or GTP/GDP [12], occurring in a wide variety of intracellular enzymes. It has been referred to as a giant anion hole [13] and has a characteristic GxxxxGKS/T consensus sequence [34]. Fig 1e shows that the P-loop has a nest incorporated within it made from five successive main chain NH groups which wrap around the β-phosphate of the nucleotide phosphate ligand, forming hydrogen bonds with the phosphate oxygen atoms. It seems probable that, in the earliest proteins, nests formed binding sites for the anionic groups of polyphosphate and acetyl phosphate (for immediate energy provision) and nucleoside polyphosphate (later, for coenzyme and RNA synthesis).

In about a half of all current protein 3D structures with iron-sulfur centres, nests with between 3 to 6 adjacent main chain NH groups are employed to help bind the Fe_2_S_2 _or Fe_4_S_4 _centres [34]. One is illustrated in Fig 1f. Like the P-loop, these features could also be evolutionary relics. Centers bound to two separate nests are not unusual. Fig 1c shows a cubane iron-sulfur center in the protein ferredoxin bound by a 6 amino acid nest. Most iron-sulfur centres are bound by cysteine thiol groups. In the earliest stages of evolution it seems probable the places of the cysteine thiol groups were occupied by organic sulphides from the hydrothermal fluid [11,42], and that cysteine binding was a later development. Nanocrystals of FeS are assembled from [2Fe-2S] rhombs [14]. The first step in growth to a potential active center is the formation of [Fe_2_S_2_] and [Fe_4_S_4_] clusters. At this stage hydrothermal thiols (RS-) tend to inhibit further growth as they sequester the rhombs as [Fe_2_S_2_(SR)_4_]^1-/2- ^and the cubanes as [Fe_4_S_4_(SR)_4_]^2-/3- ^in what may be termed "protoferredoxins" [11,43]. The sulfur atoms confer an overall negative charge to the complexes, complementing the δ+ on the NH hydrogen atoms of the nests. It seems likely nickel-bearing FeS clusters are sequestered similarly.

### d. Amyloid

Amyloid is the name given to characteristic insoluble proteinaceous fibres that are resistant to detergents and proteases. Amyloid is thought to be the primary cause of many diseases, called amyloidoses, including Alzheimer's, Parkinsons', Type II diabetes and CJD. The amyloid of prions, as in CJD, is special in being highly infective because their fibres propagate as well as being self-templating [44]. In each of these diseases, a particular protein slowly becomes converted into amyloid, giving rise to the typical late onset condition. It appears, however, that amyloid is more than an aberrant form of certain proteins produced in diseases but rather a major alternative form of most, if not all, proteins. A few native proteins do form amyloid naturally for functional reasons, such as some spider silk and bacterial surface fibrils, but these are the exception. The majority of native proteins fold to form complex, but well defined and soluble, three-dimensional structures often with α-helices and relatively small clumps of β-sheet (1–4 sheets, each clump having only 10–300 amino acids). However, most of these proteins left in aquaeous solution away from organisms eventually turn into amyloid. It seems that evolutionary pressure has ensured the majority of native proteins produced in organisms avoid turning into amyloid *in vivo*.

**Figure 2:**
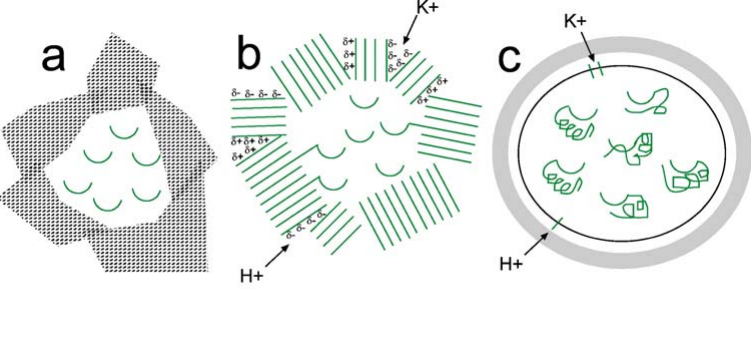
**A scheme for cell evolution**. Polypeptides are in green. **a) **The earliest cells, formed at cool hydrothermal vents: a leaky iron sulfide membrane, its metabolism assisted by phosphate and FeS-binding intracellular peptide nests shown as semicircles. **b) **More advanced cells, but now superceded: an amyloid peptide membrane shown as a mixture of apolar β-sheet and polar α-sheet; a semipermeable membrane with H+ or K+ channels at the edges of α-sheets as in Fig a stabilises the environment; intracellular nests also occur, some attached to amyloid. **c) **Current cells: a phospholiplid or etherlipid membrane plus, often, an outer cell wall. Ion channels resembling those of the earlier α-sheet are visible in the integral membrane transporter proteins. Nests occur as part of proteins.

The insoluble fibres of mature amyloid are thought to consist of stacks of say hundreds of β-sheets each with hundreds of long β-strands. Its size differentiates it from the β-sheet of soluble proteins. A small portion of a β-sheet is in Fig 1i. Current work [45,46] favours parallel β-sheet, with the in-register strands perpendicular to the fibre axis. A feature making amyloid specially suitable in early evolution was shown [47] by randomly shuffling yeast prion domains and finding they still form amyloid. It seems the amino acid composition rather than the sequence is all that is needed to form amyloid. Amyloid three-dimensional structure is simpler than the complicated arrangements of native proteins, yet, because it is sticky and does not crystallise easily, precise knowledge about its structure has been difficult to gather and still presents problems for scientists. However, in early evolution this stickiness of amyloid is likely to have been useful in forming protective insulating layers, so is a plausible candidate for the material of the first proper organic membranes, as has been suggested [48-50]. This idea is illustrated in Fig 2b.

### e. α-sheet

Certain short, non-native, polypeptides form a substance called α-sheet [51-54]. Conformationally, a single polypeptide chain of it is another example of an enantiomeric polypeptide and, as seen in Fig 1g, looks like a flattened version of the nest. Typical φ, ψ angles are -60°, -60°; 60°, 60°. Like nests, polypeptides consisting of regularly-repeating D and L amino acids favour this conformation. Multiple-stranded α-sheet resembles β-sheet, except that it is polar. One edge has a row of δ- charges, and the opposite edge has a row of δ+ charges, as in Fig 1l.

The toxic feature of amyloid in diseases is not the mature amyloid but a soluble precursor. Computer simulations by Armen *et al*. [55] give rise to the suggestion this intermediate is composed of α-sheet. Peptide plane flipping provides an easy way for α-sheet to be converted into the β-sheet of mature amyloid. Although α-sheet is rare in native proteins, peptide plane flipping of this sort occurs commonly [56], providing support for this amyloid formation pathway. The special charge distribution mentioned may underlie its toxicity on the one hand and its ability to polymerize on the other.

The rarity of α-sheet in native proteins compared to its relative stability and ease of formation in small polypeptides is striking. In any case, from the expected φ, ψ angles it would be expected to be more common than it is in native proteins. This apparent anomaly could be explained [56,57,45] by it being so toxic as an amyloid precursor that natural selection has eliminated most proteins that form α-sheet. Overall, whether or not α-sheet does prove to be the main precursor to amyloid, it seems likely it was a common conformation of the earliest peptides.

### f. Cation, proton and water channels

One problem when considering the nature of early membranes is that the constituent materials are either too permeable or too impermeable to ions and water molecules. What is needed is a semi-permeable membrane, the traditional name for the plasma membranes of existing living cells.

**Figure 3:**
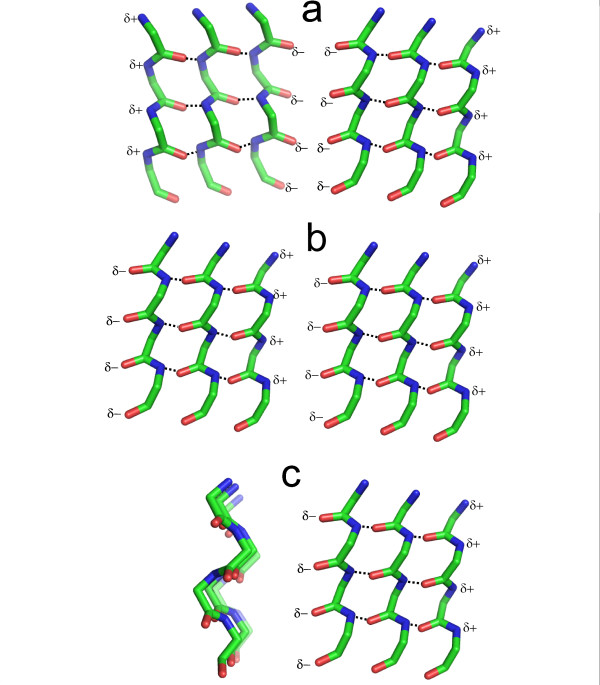
**Arrangements of α-sheet blocks**. For each block only one sheet is shown. **a) **is to be compared with the potassium channel of Fig 1j; The two adjacent blocks of α-sheet are of opposite polarity such that two rows of adjacent δ-atoms arise with potential for forming a cation channel. **b) **shows two blocks of adjacent α-sheet with the same polarity; they are liable to simply form a larger block of α-sheet. **c) **shows two α-sheet blocks with their sheets at right angles; the single row of δ-atoms seen has the potential to form a water channel as in aquaporin in Fig 1k, or a proton channel. Note the characteristic zigzag shape of the α-strands in this view. Membranes made of mixtures of α- and β-sheet blocks are also illustrated in Fig 2b.

Although α-sheet with hydrogen bonds between strands is rare in native proteins in their natural state, polypeptides with the same conformation but not arranged as α-sheet do occur in some integral membrane proteins. A group of four strands four amino acids long line the selectivity filter of the potassium channel such that the main chain carbonyl oxygen atoms form a cation channel [58,59], as shown in Fig 1j. A feature of this sort between blocks of α-sheet as in Fig 3a might have been used for cation transport early in evolution. In the water-transporting protein aquaporin a single strand with the same conformation lines one side of its water channel [60]. The row of main chain carbonyl oxygen atoms in this strand binds the δ+ protons of a corresponding row of water molecules in the channel, as in Fig 1k. Such channels can transport either protons (via a so-called proton wire) or water molecules, depending on the organisation. Aquaporin has two apparently similar but opposing channels arranged head to head such that water, rather than proton, transport is favored.

These observations, deriving from the polarity of α-sheet, lead to the idea that the edges of randomly arranged blocks of otherwise impermeable α-sheet would be likely to spontaneously form either proton, water or cation channels. In a membrane α-sheet has to be in one polarity or the other. Blocks of sheet that grow independently may happen to meet another block so that the electrostatic interaction between them, δ+ to δ-, is favorable. In this case they combine to form a larger sheet as in Fig 3b. However, if they meet in the δ- to δ- orientation, a potential cation channel is formed as in Fig 3a, whereas, if they meet side-on as in Fig 3c, a proton or water channel is liable to be formed. These effects are also indicated in Fig 2b.

### g. Metal-peptide amide nitrogen complexes

Copper, nickel, cobalt and iron cations readily complex with peptides in alkaline solutions by binding to successive main chain amide nitrogen atoms [61]. Amide NH protons are displaced by the metal during the formation of these tight complexes in which the main chain atoms adopt a flat conformation (φ = 180°, ψ = 0°), as in Fig 1h. Such complexes, with up to four amide protons substituted by metals, also occur naturally within a number of native proteins (prion protein of CJD) and enzymes (acetyl CoA synthase), sometimes at active sites. The Ni-tetraglycine complex of Fig 1h[62] exhibits a remarkable similarity to present day macrocyclic tetrapyrrole cofactors. Also the analogous Co-tetraglycine resembles a corrinoid group. In these types of complex the metal has octahedral coordination to four planar nitrogens with two positions free to assist in catalytic reactions. The peptide ones could thus have performed much the same catalytic functions as hemes, corrinoids and F_430_, before being largely, but not entirely, superceded by them. Coenzyme F_430 _is a nickel tetrapyrrole with the ability to bind and reduce methyl groups. As such it is an essential cofactor in two enzymes in the methanogenic branch of the acetyl-coenzyme A pathway, considered to be one of the earliest pathways at the origin of life [63,64]. The simpler, acetogenic branch of the acetyl coenzyme-A pathway involves an iron-sulfur protein with a corrinoid cofactor – a cobalt-tetrapyrrole – that transfers a methyl group to acetyl coenzyme-A synthetase [64-66].

## Conformations for protoenzymes and membranes

Table 1 summarizes the frequencies we expect, providing comparisons with conformations of native and unfolded present-day proteins. Three conformations, amyloid β-sheet, α-sheet and nests are predicted to be the commonest in early evolution, while structures where amino acids are markedly of one chirality, such as PPII and α-helix, would have been less common then than now.

An important function of the β-sheet of mature amyloid is proposed to be the coating, and partially clogging up, of pores in the submarine mineral chambers. Such sticky amyloid peptides would have constituted the beginnings of a cell membrane, combining both strength as in a cell wall and impermeability as in a cell membrane. That some kind of peptidic membrane encapsulated early microbes is suggested by the fact that the biosynthesis of the fatty acid ester membranes of the bacteria and of the isoprenoid ether membrane of the archaea clearly post-dated the differentiation of these two domains [67,68,5]. *E. coli *can produce extracellular fimbriae-like amyloidal structures [69]. These polymers colonize inert surfaces by forming biofilms. The filaments grow by addition of monomers to their free growing ends [70,71]. If amyloid formed the earliest membranes, which were then superceded by phospholipid ones, it is not unexpected that amyloid would interfere with phoshopholipid membranes in some way as indeed it does.

Another in the common category is α-sheet, an enantiomeric structure, which is observed in short polypeptides [52,54], though not in those found *in vivo*. α-sheet is suggested to be the precursor to amyloid by forming readily from unfolded peptides and converting directly into the mature β-sheet form. Given that amyloid is difficult to study by crystallography, there is a lack of biophysical techniques that reliably distinguish α- and β-sheet [54]. Indeed they may continuously interconvert. Another point about α-sheet is that the edges of this material might naturally form cation, proton or water channels through membranes. Amyloidogenic aggregates readily bind to biological and artificial phospholipid membranes [72]. They can insert themselves into the membranes altering their fluidity, and also giving rise to ion channels, resulting, it is thought, in cell toxicity [57,73]. It should be reiterated that the beguiling idea that α-sheet is the material of the prefibrillar intermediate responsible for such alterations to membranes is not yet generally accepted, but the effects of what may be an earlier type of membrane, whatever its molecular nature, on present-day membranes are clearly of interest.

Nests are are useful for ligand-binding, particularly phosphate groups and iron-sulfur centers. On the early Earth the polymerization of inorganic phosphate would have been important to the energetics of the first cells. The precursor molecules of metabolism, viz., the transition metal sulfide thiolates, the phosphates (CH_3_OPO_3_^2-^, HP_2_O_7_^3- ^and HPO_4_^2-^), and also short achiral peptides, would be retained within the metal sulfide compartments as in Fig 2a. Another peptide conformation, which, though not necessarily quantitatively large, could have been critical, are the (Fe, Ni, Co)-peptide complexes described earlier as these would have provided many of the catalytic and binding functions of present-day tetrapyrroles.

Nests, collecting on the inner walls of the initially inorganic membrane, would have spaced and insulated the active [4Fe-4S]-centers at the distances of several Å suitable for the storage and transfer of electrons. Pyro- and acetyl-phosphate could also be retained within the membrane to act as phosphorylating and/or condensing agents. In the absence of nests, active catalytic metal sulfide and pyrophosphate sites would loose their activities on hydration and/or ripening through a nanocrystalline stage. Crystalline precipitates typical of hydrothermal ore deposits [cf. [74]] would result. Alternatively they would dissolve as pH dropped in the membrane. Nests would also alter electrochemical properties by stabilizing the reduced, more than the oxidized, forms of iron-sulfur centers and thereby reduce the Eh values of these protoenzymes.

## Conclusion

We have considered, given that glycine-rich and otherwise heterochiral peptides emerged at an early stage in evolution, what polypeptide conformations might have predominated and with what functional effects. Current knowledge about protein and peptide 3D structure indicates that such peptides naturally form themselves under the prevailing conditions into nest, α-sheet, amyloid and metal-peptide structures, but less often into α-helix and polyproline type II helices. Enantiomeric structures are favored at the expense of helical conformations. The evolution scenario goes thus: Starting from a largely inorganic world, the organic products of an Fe(Ni)S protometabolism, developing in a submarine hydrothermal reactor, complicate the system in such a way as to enhance its efficiency. Acetate is considered the most likely initial product of metabolism, with amino acids and some peptide being uncommon by-products at first.

The percentage conversion of acetate from reactants CO_2_, HCOO^- ^and H_2 _is low because most of the metal sulfide had low catalytic activity. However, once significant amounts of short peptides appear the situation changes. The ability to bind and stabilise these catalysts improves the efficiency of acetate and amino acid production. There is now a virtuous circle: peptides promote Fe(Ni)S nanoclusters which in turn catalyse amino acid and peptide synthesis. In the absence of the protection offered by nests, metal sulfide and pyrophosphate clusters lose their activities on hydration, dissolution or by ripening through a nanocrystalline stage to crystalline precipitates with the relatively low surface-to-volume ratios typical of hydrothermal minerals. Metal-peptides also form, providing primitive tetrapyrrole-like groups useful for other sorts of catalysis. Next, the peptides form into sticky blocks of amyloid that clump together. Some of these clumps have spaces within them acting as cells, bounded by amyloid semi-permeable membranes as in Fig 2b. The membranes have pores within them that exert some control over the passage of cations and water, maintaining a more constant environment for the developing metabolisms. In later cells the membrane functions are taken over by membranes of phospholipid or etherlipids plus cell walls, as in Fig 2c, although key parts of the old channels remain incorporated in the ion transporters and aquaporins. Amyloid, because it interferes with the new membranes, is largely eliminated by evolution, except for a few specialized purposes, though it continues to threaten aged organisms, to which the selection process hardly applies, with ailments such as Alzheimer's. The functions of metal-peptides are taken over by tetrapyrroles. Nests, on the other hand, become embedded within proteins. Phosphate-binding P-loops and loops of some iron-sulfur binding proteins retain the traces of their previous existence.

## Competing interests

The author(s) declare that they have no competing interests.

## Reviewers' comments

### Bill Martin

This is an interesting paper pointing out the possible types of interactions that could have taken place between short heterochiral peptides and inorganic catalysts during chemical evolution. It makes several novel and worthwhile points.

### Eugene Koonin

This article continues the series of studies by Russell and coworkers on the origin of life in networks of inorganic compartments existing at hydrothermal vents. This paper focuses on primordial amino acid and peptide chemistry and attempts to infer the distribution of conformations of primordial, primarily, abiogenic peptides and small proteins. Predictably, these abiogenic peptides are thought to be heterochiral and as a result, the population of the conformational space would be very different from that in modern proteins. I am particularly interested in the authors' conjecture that abiogenic peptides, such as the anion-binding nests, could facilitate the formation of the first transmembrane potential and so could be central to the emergence of bioenergetics. Other unusual structures would contribute to the formation of the primordial membranes themselves and membrane channels, and yet others would become catalysts. By and large, I find the scenario of the participation of biogenic peptides in the emergence of biochemistry and bioenergetics outlined in this paper to be interesting and plausible. I also agree that P-loops and iron-binding loops must have been among the earliest peptide structures.

The biggest remaining question is how did the transition from the abiogenic, enantyomeric was brought about. Recently, we outlined a scenario under which the utility of amino acid and peptides in the emerging biological systems drove the evolution of translation, through the intermediate stage of peptide ligases (Wolf, Koonin, On the origin of the translation system and the genetic code in the RNA world by means of natural selection, exaptation, and subfunctionalization. Biol Direct 2007 May 31;2:14). I believe that, at least, conceptually, this line of thinking about the origin of life is remarkably well compatible with the ideas on the role of abiogenic peptides in primordial biochemistry developed by Milner-White and Russell. Perhaps, this connection could be briefly discussed in the paper.

### Nick Grishin

In a somewhat unusual review, the authors discuss potential conformations of ancient pre-biotic peptides and present hypotheses about their possible interactions and functions. While nothing in the manuscript contradicts the main principles of physics and evolution, I am left with a mixed feeling. Why would the conformation of early peptides (obviously prebiotic, since they are DL mixes) be of interest when we are not sure whether the hypothesis about the importance of pre-biotic peptides has any merit? We know that chemical, pre-biotic peptide formation is not an easy process (e.g. experimental papers the authors cite), so there is no basis to believe that polypeptides of reasonable length were any abundant, and that they have played a significant role early on in prebiotic evolution. It is equally not clear why regular DLDLDL mixtures should form in pre-biotic conditions. Thus, it seems to me that prior to addressing the question about conformations, it would be nice to discuss these questions. The hypothesis that polypeptides were rare and not significance in prebiotic evolution makes better sense to me. The fact that peptides are so common and important today, does not imply that they were significant early on. In any case, this is such an imprecise area of research, that it would be good to describe any alternative scenarios and potential pitfalls, other hypotheses and rationalizations. Presentation of these alternatives would make for a more interesting reading.
